# Cancer/Testis antigens as potential predictors of biochemical recurrence of prostate cancer following radical prostatectomy

**DOI:** 10.1186/1479-5876-9-153

**Published:** 2011-09-14

**Authors:** Takumi Shiraishi, Naoki Terada, Yu Zeng, Takahito Suyama, Jun Luo, Bruce Trock, Prakash Kulkarni, Robert H Getzenberg

**Affiliations:** 1 James Buchanan Brady Urological Institute, Department of Urology, The Johns Hopkins University School of Medicine, Baltimore, MD 21287, USA; 2 Department of Urology, Graduate School of Medical Science, Kyoto Prefectural University of Medicine, Kyoto 602-0841, Japan; 3 Department of Urology, Graduate School of Medicine, Chiba University, Chiba 260-8670, Japan; 4 Sidney Kimmel Comprehensive Cancer Center, Department of Oncology, The Johns Hopkins University School of Medicine, Baltimore, MD 21287, USA; 5 Department of Pharmacology and Molecular Sciences, The Johns Hopkins University School of Medicine, Baltimore, MD 21287, USA

## Abstract

**Background:**

The Cancer/Testis Antigens (CTAs) are an important group of proteins that are typically restricted to the testis in the normal adult but are aberrantly expressed in several types of cancers. As a result of their restricted expression patterns, the CTAs could serve as unique biomarkers for cancer diagnosis/prognosis. The aim of this study was to identify promising CTAs that are associated with prostate cancer (PCa) recurrence following radical prostatectomy (RP).

**Methods:**

The expression of 5 CTAs was measured by quantitative multiplex real-time PCR using prostate tissue samples obtained from 72 patients with apparently clinically localized PCa with a median of two years follow-up (range, 1 to 14 years).

**Results:**

The expression of CTAs namely, CEP55, NUF2, PBK and TTK were significantly higher while PAGE4 was significantly lower in patients with recurrent disease. All CTAs with the exception of TTK were significantly correlated with the prostatectomy Gleason score, but none were correlated with age, stage, or preoperative PSA levels. In univariate proportional hazards models, CEP55 (HR = 3.59, 95% CI: 1.50-8.60), p = 0.004; NUF2 (HR = 2.28, 95% CI: 1.11-4.67), p = 0.024; and PAGE4 (HR = 0.44, 95% CI: 0.21-0.93), p = 0.031 were significantly associated with the risk of PCa recurrence. However, the results were no longer significant after adjustment for prostatectomy Gleason score.

**Conclusions:**

To our knowledge, this is the first study to identify CTAs as biomarkers that can differentiate patients with recurrent and non-recurrent disease following RP and underscores its potential impact on PCa prognosis and treatment.

## Background

Prostate cancer (PCa) is the most common malignancy and the second leading cause of male cancer-related death in the U.S. [1]. The introduction of serum prostate-specific antigen (PSA) has dramatically altered and benefited the initial diagnosis of men with PCa. However, the widespread use of PSA testing has resulted in over-detection and over-treatment of potentially indolent disease [2]. Therefore, there is a dire need for better tools to differentiate men that will benefit from definitive therapy from those in whom an active surveillance program may be appropriate. Therefore, in addition to the currently utilized clinical information, we also need better markers that are able to identify men that need definitive or adjuvant therapy after radical prostatectomy (RP).

The Cancer/Testis Antigens (CTAs) are a group of proteins that in the normal adult are typically restricted to the testis but are aberrantly expressed in several types of cancers [3]. Although CTAs are generally highly expressed in testis, these genes exhibit heterogeneous expression profiles, and can be classified into testis-restricted, testis/brain-restricted, and testis-selective groups of genes that show additional expression in somatic tissues [4]. Furthermore, the CTAs can be broadly divided into two groups based on their chromosomal location: the CT-X antigens located on the × chromosome and non-X CT antigens located on various autosomes. In the normal testis, the CT-X genes are generally expressed in the spermatogonia, the proliferating germ cells [5]. On the other hand, expression of non-X CT genes appears more dominant in the later stages of germ-cell differentiation, such as in spermatocytes [5]. In several types of cancers, individual CTAs are often associated with advanced disease with poorer outcomes in addition to their specific expression patterns [6-11]. Additionally, we recently demonstrated that there is a remarkable stage-specific expression of the CTAs in PCa [12]. These observations raised the possibility that the CTAs might serve as unique biomarkers that could potentially be used to distinguish men with aggressive disease who need treatment from men with indolent disease not requiring immediate intervention in PCa.

In this study, by mining publicly available microarray data in conjunction with data on CTA expression that we obtained using a custom CT microarray [12], we explored CTAs that were associated with disease recurrence following RP.

## Methods

### Clinical samples

Prostate tissue specimens from 72 clinically localized PCa cases with follow-up data (29 without recurrence and 43 with recurrence) and 17 clinically localized PCa without follow-up data were collected and frozen at the time of radical prostatectomy (RP), from 1993 to 2007, at the Johns Hopkins Hospital. Twenty histologically normal samples of prostate tissues were collected from cancer-free regions of surgical specimens obtained at the time of RP. The tissue specimens were processed as described previously before RNA extraction [13]. OCT-embedded frozen tissue blocks were manually trimmed to enrich the content of target tissue lesions (e.g., normal or cancer) prior to sectioning and RNA extraction. For tumor samples, tumor cells made up more than 70% of the tissue content (calculated by averaging the % tumor content in the first and last sections) in all cases [14]. Metastatic PCa tissues (n = 21) were collected from soft tissue metastasis of patients who died from PCa, as part of the Johns Hopkins Autopsy Study of lethal PCa [15]. Autopsy RNA specimens had high quality as assessed by RNA index number (RIN), and were prepared and processed as previously described [16,17]. Twenty normal prostate tissues, 17 localized PCa without follow-up data and 21 metastatic PCa samples were used to validate the microarray data obtained from database (Additional file 1). Seventy two localized PCa samples were used to investigate whether selected CTAs were associated with disease recurrence following RP. The use of surgical and autopsy specimens for molecular analysis was approved by the Johns Hopkins Medicine Institutional Review Boards. Biochemical recurrence was defined as a postoperative elevation of serum PSA (0.2 ng/ml or greater) after RP [18,19]. The endpoint of the follow-up in this study was the time to biochemical recurrence.

### Quantitative singleplex and multiplex real-time PCR

First strand cDNA was made from 0.5 μg RNA using iScript cDNA Synthesis Kit (Bio-Rad Laboratories, Inc., Hercules, CA) following the manufacturer's protocol in a total volume of 20 μl. All primers and probes (Additional file 2) were obtained from Integrated DNA Technologies (Coralville, IA). Quantitative singleplex and multiplex real-time PCR were carried out with the CFX96 Real-Time PCR Detection System (Bio-Rad Laboratories, Inc.). The singleplex PCR reactions were performed with 0.2 μl of cDNA template in 25 μl of reaction mixture containing 12.5 μl of iQ SYBR Green Supermix (Bio-Rad Laboratories, Inc.) and 0.25 μmol/L each primer. PCR reaction was subjected to hot start at 95°C for 3 minutes followed by 45 cycles of denaturation at 95°C for 10 seconds, annealing at 60°C for 30 seconds, and extension at 72°C for 1 minute. Quantitative multiplex real-time PCR reaction was carried out with 0.2 μl of cDNA template in 25 μl of reaction mixture containing 12.5 μl of iQ Multiplex Powermix (Bio-Rad Laboratories, Inc.), 0.3 μmol/L each primer and 0.2 μmol/L probe. The reaction was subjected to hot start at 95°C for 3 minutes followed by 45 cycles of denaturation at 95°C for 10 seconds, and annealing and extension at 60°C for 60 seconds. Amplification of β-actin was used as a control for RNA integrity for all samples. We confirmed that almost identical results were obtained when using TATA binding protein as a reference gene instead of β-actin (data not shown). A reference RNA from pooled cells and prostate tissue were included on each plate to provide standardization across PCR plates. Analysis and fold-change differences were determined using the comparative CT method. Fold change was calculated from the ΔΔCT values with the formula 2 ^-ΔΔCT^.

### Statistical analysis

CTA expression was normalized by dividing each individual value by their respective β-actin value. Distributions of normalized CTAs were compared among categories of prognostic factors using the Wilcoxon test (for binary factors) or Kruskal-Wallis test (for factors with more than 2 categories). Correlations between the normalized CTAs and other continuous variables were evaluated by linear regression and the Pearson correlation coefficient. Normalized CTAs were dichotomized at the median of the distribution among prostate cancer patients who did not have a recurrence. Risk of recurrence associated with CTAs was evaluated in univariate and multivariable proportional hazards models. All statistical analyses were performed with SAS v9.2 (SAS Institute, Cary, NC).

## Results

### Selection of candidate CTAs

By mining publicly available microarray data from the Gene Expression Omnibus http://www.ncbi.nlm.nih.gov/geo in conjunction with our own data [12], we first selected candidate CT-X and non-X CTAs for investigation. As described in Additional file 1, the CT-X antigens (SSX2, CSAG2, MAGEA2, and MAGEA12) were highly up-regulated only in metastatic PCa but were not expressed in benign and primary PCa. The highest expression of non-X CT antigens (CEP55, NUF2, PBK and TKK) was seen in metastatic PCa similar to the CT-X antigens; however the non-X CT antigens were also up-regulated in primary PCa compared to benign prostate disease. Interestingly, a member of the CT-X antigen family, PAGE4, was highly expressed in benign and primary PCa but not in metastatic PCa.

**Figure 1:**
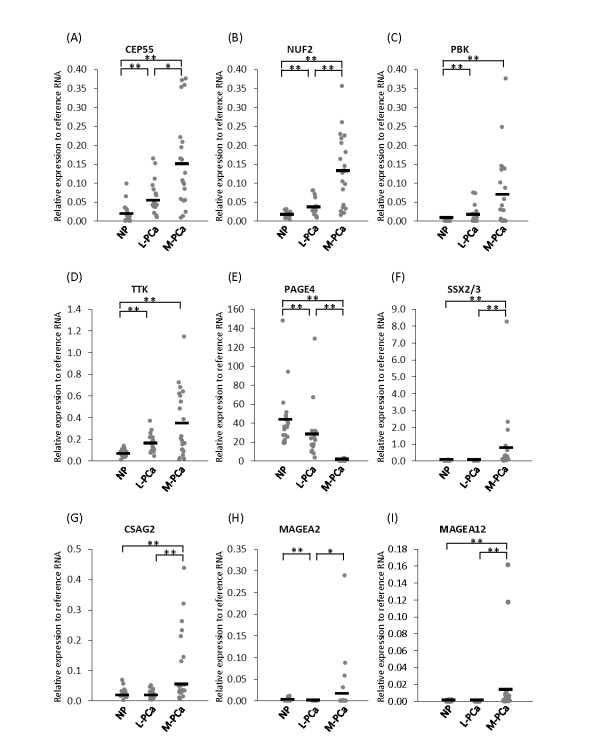
**Cancer/Testis Antigen (CTA) expression in prostate tissues**. CTA expression in normal prostate tissue (NP) (n = 20), clinically localized prostate cancer (L-PCa) (n = 17) and metastatic prostate cancer (M-PCa) (n = 21). (A) CEP55, (B) NUF2, (C) PBK, (D) TTK, (E) PAGE4, (F) SSX2/3, (G) CSAG2, (H) MAGEA2, (I) MAGEA12. * *p *< 0.05, **p < 0.01.

To validate the microarray data, we performed quantitative real-time PCR using samples from normal prostate (n = 20), clinically localized PCa (n = 17) and metastatic PCa (n = 21) (Figure 1). Consistent with the microarray data, all of the CTAs with the exception of PAGE4 were highly expressed in metastatic disease. Furthermore, the expression of four non-X CT antigens (CEP55, NUF2, PBK and TKK) was significantly different between normal prostate and clinically localized PCa. In contrast, there were no significant differences between normal prostate and clinically localized PCa for the four CT-X antigens namely, SSX2, CSAG2, MAGEA2, and MAGEA12. However, PAGE4 showed the highest expression in normal prostate but was significantly down-regulated in metastatic samples.

The aim of this study was to determine if CTAs are associated with disease recurrence after radical prostatectomy (RP). Since the following five CTAs, CEP55, NUF2, PBK, TKK and PAGE4 showed differential expression in both clinically localized PCa and metastatic PCa compared to normal prostate, we selected these five CTAs for developing a multiplex PCR assay that has the potential to stratify patients with and without PCa recurrence after RP.

### Association of CTA expression with recurrence in radical prostatectomy patients

**Figure 2:**
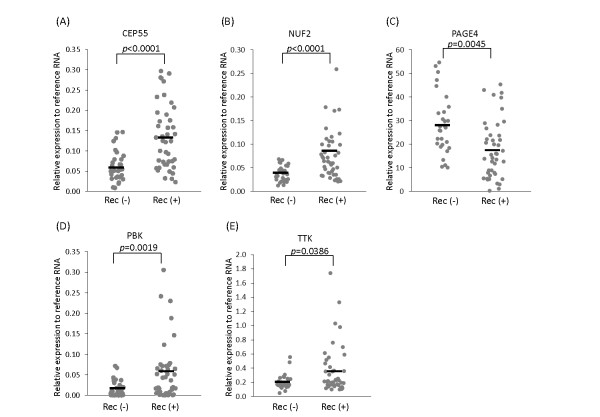
**Cancer/Testis Antigen (CTA) expression in recurrent and non-recurrent prostate cancer**. CTA expression in clinically localized prostate cancer with recurrence (Rec (+)) (n = 43) and without recurrence (Rec (-)) (n = 29). (A) CEP55, (B) NUF2, (C) PAGE4, (D) PBK, (E) TTK.

In order to characterize the selected CTAs, we analyzed 72 samples from PCa patients who underwent radical prostatectomies for their disease (29 patients without recurrence and 43 patients with recurrence). Characteristics of the prostatectomy patients are described in Table 1. In this cohort, 36% of the patients had Gleason 8-10 and a fairly large fraction (60%) experienced biochemical recurrence within a median of 1 year follow-up (range, 1 to 8 years), indicating that this cohort was a very high risk cohort. As shown in Figure 2A-D, CEP55 (p < 0.0001), NUF2 (p < 0.0001), PBK (p = 0.0045) and TTK (p = 0.0386) were significantly higher in prostate samples from patients with recurrent disease. In contrast, PAGE4 (p = 0.0019) was significantly lower in the same patients (Figure 2E). However, there was no significant difference in the CT-X antigens (SSX2, CSAG2, MAGEA2, and MAGEA12) between patients with or without recurrence (data not shown).

**Table 1:** Characteristics of the prostatectomy patients evaluated (1993-2007)

	Men without Recurrence (n = 29)	Men with Recurrence (n = 43)	*P *Value
Follow-up period (years)			
Mean ± SD	5.3 ± 3.1	1.7 ± 1.4	
Median	5	1	
Interquartile range	4	1	
Range	1-14	1-8	
Age at surgery (years)			
Mean ± SD	54.8 ± 6.7	59.4 ± 6.9	< 0.01
Median	54	61	
Range	43-68	41-71	
Preoperative PSA (ng/ml)			
Mean ± SD	7.9 ± 6.7	12.9 ± 8.8	< 0.01
Median	6.1	10.7	
Range	1.7-31.6	3.7-37.4	
Pathological Gleason sum, n (%)			
≤ 6	20 (70.0)	1 (2.3)	< 0.001
7 (3 + 4)	3 (10.3)	13 (30.2)	
7 (4 + 3)	2 (6.9)	7 (16.3)	
8-10	4 (13.8)	22 (51.2)	
Pathological stage, n (%)			
OC (EPE-, SV-, LN-)	10 (34.5)	2 (4.7)	< 0.001
EPE+ (SV-, LN-)	16 (55.2)	20 (46.5)	
SV+ (EPE+/-, LN-)	3 (10.3)	9 (20.9)	
LN+ (EPE+/-, SV+/-)	0	12 (27.9)	

PSA, prostatic-specific antigen; OC, organ-confined; EPE, extra-prostatic extension; SV, seminal vesicle; LN, lymph node; CTAs, Cancer/Testis Antigens

### Association of CTA expression with clinicopathological parameters

We next compared CTA expression patterns with prognostic variables such as the age of patients, preoperative PSA, Gleason grade, and tumor stage. All of the CTAs with the exception of TTK, were significantly correlated with the prostatectomy Gleason score (CEP55; p = 0.0057, NUF2; p = 0.0051, PBK; p = 0.0295, PAGE4; p = 0.0236); however none were correlated with age, preoperative PSA and tumor stage.

### Association of CTA expression with biochemical-free survival

**Figure 3:**
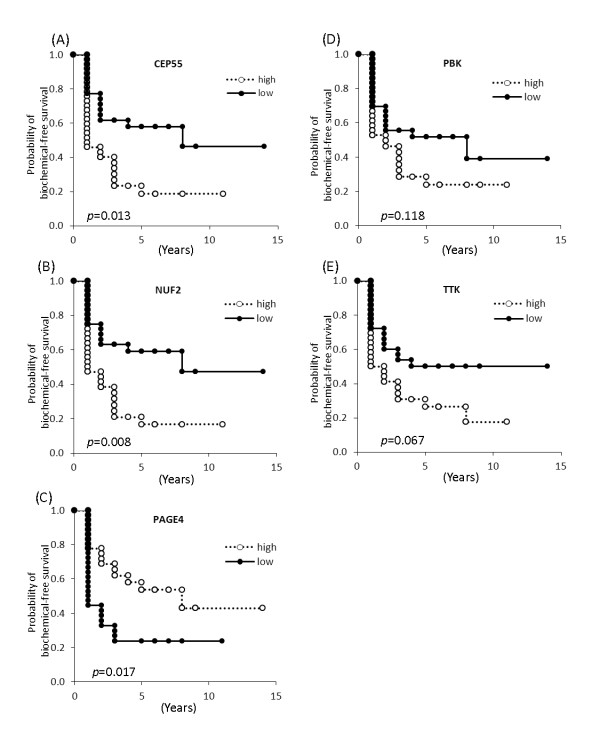
**Kaplan-Meier analyses**. Kaplan-Meier curves showing biochemical recurrence-free survival against time after radical prostatectomy stratified by the mRNA expression of (A) CEP55, (B) NUF2, (C) PAGE4, (D) PBK, and (E) TTK (high versus low groups dichotomized by median value).

Kaplan-Meier analyses were performed with dichotomized CTA expression (according to the median) and the time parameter of biochemical-free survival. Higher expression of CEP55 and NUF2 was significantly correlated with shorter biochemical recurrence-free time (Figure 3A, B). In contrast, higher expression of PAGE4 was significantly correlated with longer biochemical recurrence-free time (Figure 3C). In univariate proportional hazards models, CEP55 (HR = 3.59, 95% CI: 1.50-8.60), p = 0.004; NUF2 (HR = 2.28, 95% CI: 1.11-4.67), p = 0.024; and PAGE4 (HR = 0.44, 95% CI: 0.21-0.93), p = 0.031 were significantly associated with the risk of PCa recurrence (Table 2). Multivariable proportional hazards models evaluated the effect of established prognostic factors including age, PSA, pathologic stage, and prostatectomy Gleason score; only the latter was statistically significant in the multivariable model. Each CTA was tested individually in a multivariable model that also adjusted for prostatectomy Gleason score (Table 3). None of the CTAs retained statistical significance after adjustment for prostatectomy Gleason score, indicating that at least in this cohort the CTAs are not independent predictors of biochemical recurrence.

**Table 2:** Univariate proportional hazards models of risk of biochemical recurrence after prostatectomy associated with established prognostic factors and individual CTAs*

Variable	HR (95% CI)	*P *Value
Age (years)	1.04 (0.999, 1.09)	0.054
PSA (ng/ml)	1.04 (1.004, 1.07)	0.028
Prostatectomy stage		
Organ confined	1.0	ref
Extraprostatic extension	4.53 (1.05, 19.47)	0.042
Seminal vesicle involvement	7.49 (1.60, 35.10)	0.011
Lymph node metastases	10.81 (2.37, 49.23)	0.002
Prostatectomy Gleason score		
≤ 6	1.0	ref
7 (3 + 4)	26.83 (3.48, 206.73)	0.002
7 (4 + 3)	39.01 (4.65, 327.17)	0.0007
8 to 10	35.01 (4.64, 264.19)	0.0006
CTAs		
CEP55	3.59 (1.50-8.60)	0.004
NUF2	2.28 (1.11-4.67)	0.024
PAGE4	0.44 (0.21-0.93)	0.031
PBK	1.78 (0.92-3.43)	0.085
TTK	1.55 (0.82-2.94)	0.179

CTAs, Cancer/Testis Antigens; HR, hazard ratio; CI, confidence interval

* CTAs were dichotomized at the median of the value normalized to actin in the non-recurrent group. Hazard ratios represent comparison of values greater than or equal to the median vs. values less than the median.

**Table 3:** Multivariable proportional hazards models of risk of biochemical recurrence after prostatectomy associated with prostatectomy Gleason score and individual CTAs*

Variable	HR (95% CI)	*P *Value
CEP55		
CEP55	1.85 (0.76, 4.53)	0.177
Prostatectomy Gleason score		
≤ 6	1.0	ref
7 (3 + 4)	22.07 (2.82, 172.74)	0.003
7 (4 + 3)	28.90 (3.34, 250.28)	0.002
8 to 10	29.25 (3.82, 224.08)	0.001
NUF2		
NUF2	1.73 (0.83, 3.63)	0.145
Prostatectomy Gleason score		
≤ 6	1.0	ref
7 (3 + 4)	25.04 (3.24, 193.72)	0.002
7 (4 + 3)	31.21 (3.66, 266.07)	0.002
8 to 10	33.31 (4.40, 251.92)	0.0007
PAGE4		
PAGE4	0.70 (0.33, 1.49)	0.351
Prostatectomy Gleason score		
≤ 6	1.0	ref
7 (3 + 4)	24.53 (3.16, 190.61)	0.002
7 (4 + 3)	32.90 (3.83, 282.99)	0.002
8 to 10	32.73 (4.32, 248.21)	0.0007
PBK		
PBK	1.40 (0.72, 2.73)	0.321
Prostatectomy Gleason score		
≤ 6	1.0	ref
7 (3 + 4)	26.41 (3.42, 203.76)	0.002
7 (4 + 3)	36.04 (4.27, 304.10)	0.001
8 to 10	33.30 (4.40, 252.02)	0.0007
TTK		
TTK	1.27 (0.67, 2.42)	0.470
Prostatectomy Gleason score		
≤ 6	1.0	ref
7 (3 + 4)	25.56 (3.31, 197.50)	0.002
7 (4 + 3)	38.46 (4.59, 322.31)	0.0008
8 to 10	33.55 (4.44, 253.06)	0.0007

CTAs, Cancer/Testis Antigens; HR, hazard ratio; CI, confidence interval

* CTAs were dichotomized at the median of the value normalized to actin in the non-recurrent group. Hazard ratios represent comparison of values greater than or equal to the median vs. values less than the median.

## Discussion

If we acknowledge that we are currently 'over-detecting' PCa as a result of PSA-based screening, then there is a dire need to develop new biomarkers to improve the over-detection (and consequently, over-treatment) of potentially indolent disease. Unfortunately, currently available biomarkers are unable to discern indolent from aggressive disease.

Recently, we demonstrated the potential of the CTAs to serve as novel biomarkers for differentiating organ-confined PCa and metastatic PCa [12]. In this study, we identified several CTAs that are differentially expressed in normal prostate, localized PCa, and metastatic PCa tissues. From this initial set, we identified five CTAs that are differentially expressed between patients with and without disease recurrence after RP. Furthermore, we demonstrated that 3 of these CTAs (CEP55, NUF2 and PAGE4) were significantly associated with the risk of PCa recurrence. Therefore, these CTAs may represent good biomarker candidates to predict disease recurrence following RP. To our knowledge, this is the first study analyzing the value of expression levels of CEP55, NUF2 and PAGE4 in localized PCa treated by RP.

According to the classification of CTAs defined by Hoffman et al. [4], CEP55 and NUF2 are considered to be classified as a testis-selective group of genes that show additional expression in somatic tissues in addition to the testis (Additional file 3). CEP55 is a centrosomal protein that plays an essential role in G2/M cell cycle [20,21] while human NUF2 encodes a protein that is highly similar to yeast Nuf2. Yeast Nuf2 is a component of a conserved protein complex associated with the centromere and plays a regulatory role in chromosome segregation [22]. Human NUF2 is also found to be associated with centromeres of mitotic HeLa cells, which suggests that this protein is a functional homolog of yeast Nuf2 [23]. Thus, higher expression of CEP55 and NUF2 in prostate samples from patients with recurrent disease after RP might be associated with an inappropriate regulation of G2/M transition of cell cycle.

Unlike a typical CT-X antigen that is predominantly expressed in the testis, PAGE4 exhibits a unique expression pattern (Additional file 3). It is reported that PAGE4 is expressed both in normal prostate and prostate cancer, but is also expressed in other male and female reproductive tissues including testis, fallopian tube, placenta, uterus, and uterine cancer [24]. Furthermore, PAGE4 is known to be up-regulated in symptomatic benign prostatic hyperplasia (BPH) [25] and in PCa [26]. Inconsistent with the previous data, we demonstrated that PAGE4 showed higher expression in normal prostate compared to localized PCa. We also showed that PAGE4 was significantly down-regulated in metastatic PCa compared to localized PCa. Considering these results, we hypothesized that PAGE4 might play an important role especially in the early stage of PCa and not be required for disease progression. Because this patient cohort was very high risk, there is a possibility that samples of localized PCa used in this study might have more aggressive phenotype (high metastatic potential) than those used in other studies. Furthermore, the normal prostate tissues used in this study were histologically normal prostate samples from surgical specimens with prostate cancer. Normal prostate tissues obtained from PCa patients might have higher expression of PAGE4 compared to normal prostate tissues from healthy donor. However, further examination is required to validate the down-regulation of PAGE4 with disease progression of PCa.

It is reported that PCa exhibits moderate expression levels of CTAs with 30% of the CTAs transcripts having an expression frequency of greater than 20% [3]. In this study, we analyzed the expression levels of CTAs using quantitative real-time PCR, as opposed to the end-point RT-PCR commonly used in the previous studies. We observed that the CTAs were expressed in all of the samples studied, although the expression levels varied significantly. Further study is required to investigate the frequency of these CTAs in PCa using immunohistochemical analysis, especially in order to examine the potential of these CTAs as immunotherapeutic targets for PCa.

Several points in this report warrant emphasis. First, in this study, we show that all CTAs with the exception of TTK were significantly correlated with prostatectomy Gleason score, but none were correlated with age, preoperative PSA and tumor stage. Although Gleason score is believed to be one of the strongest predictors of recurrence, it tends to be subjective. As described, we developed quantitative multiplex real-time PCR assays to determine CTA expression that in future should provide consistent and quantitative data across institutions. Therefore, these have the potential to be a 'molecular' Gleason's score. Second, cancer immunotherapy is emerging as a promising modality for castration- and chemotherapy-resistant PCa [27]. Indeed, many CTAs are immunogenic and their use as therapeutic cancer vaccines is being evaluated. In fact, CEP55 and NUF2 have already been shown to be useful targets for immunotherapy [28-30]. Thus, a CTA-based biomarker can provide information not only for predicting disease recurrence, but also suggesting treatment options, that can be personalized to the patient. Third, one of the problems in developing reliable biomarkers for PCa is heterogeneity. Therefore, a combination of markers is more likely to provide better predictive value rather than a single marker. With the use of a multiplex qPCR assay as the detection platform, there is the potential to expand the test to include additional transcripts which can enhance assay performance.

On the other hand, there are several limitations to the present study as well. The patient number was limited and these patients were not consecutively and prospectively collected for this study. Furthermore, we used a frozen tissue cohort in which selection bias existed as a result of selection of specimens with large volume tumors appropriate for frozen tissue collection. In fact, the patients in this cohort had high Gleason grade (> 8) (36%), extra-prostatic extension (50%), seminal vesicle involvement (17%), and lymph node metastasis (17%), resulting in a high biochemical recurrence rate (60%), not reflecting contemporary newly screened RP population [18,31,32]. Thus, we evaluated a set of CTAs in such a high risk cohort due to the selection bias. Therefore, larger and contemporary cohorts presenting newly diagnosed RP population across multiple institutions are required for further validation of these results.

## Conclusion

The present study has demonstrated the potential of the CTAs as a prognostic marker for patients after RP. Taking it into consideration that currently there is no biomarker available to discern indolent from aggressive disease, these results appear promising although, additional studies will be required to validate the results in a larger setting.

## Competing interests

A provisional patent application covering this invention has been filed on behalf of PK, TS (Takahito Suyama), TS (Takumi Shiraishi) and RHG by Johns Hopkins University.

## Authors' contributions

TS (Takumi Shiraishi), NT and YZ contributed to the study design, experimental work and manuscript preparation. TS (Takahito Suyama) and JL contributed to sample collection. BT performed the statistical analysis. PK and RHG conceived of the study, and participated in its design and coordination and helped in drafting the manuscript. All authors read and approved the final manuscript.

## Supplementary Material

Additional file 1 **Supplemental Table 1**. Differential expression of the Cancer/Testis Antigens in benign prostate, primary prostate cancer, and metastatic prostate cancer. This file contains the expression data of the Cancer/Testis Antigens in benign prostate, primary prostate cancer, and metastatic prostate cancer from the Gene Expression Omnibus http://www.ncbi.nlm.nih.gov/geo. Click here for file

Additional file 2 **Supplemental Table 2**. PCR primer and probe sequences for quantitative singleplex and multiplex real-time PCR. This file contains PCR primer and probe sequences used in this study. Click here for file

Additional file 3 **Supplemental Figure 1**. Gene expression microarray data of NUF2, CEP55, PBK, TTK and PAGE4 in various normal tissues. Gene expression data from various normal tissues for (A) NUF2, (B) CEP55, (C) PBK, (D) TTK and (E) PAGE4 were extracted from microarray study [33] annotated in the Oncomine database [34]. Click here for file
