# Supplementary material for: Cancer/Testis antigens as potential predictors of biochemical recurrence of prostate cancer following radical prostatectomy
Source: J Transl Med. 2011 Sep 14;9:153. doi: 10.1186/1479-5876-9-153 (PMC3184272; doi:10.1186/1479-5876-9-153)
Supplement: Additional file 1 — Supplemental Table 1. Differential expression of the Cancer/Testis Antigens in benign prostate, primary prostate cancer, and metastatic prostate cancer. This file contains the expression data of the Cancer/Testis Antigens in benign prostate, primary prostate cancer, and metastatic prostate cancer from the Gene Expression Omnibus http://www.ncbi.nlm.nih.gov/geo. [file 1479-5876-9-153-S1.DOC]

Supplemental Table 1. Differential expression of the Cancer/Testis Antigens in benign prostate, primary prostate cancer, and metastatic prostate cancer

| **GEO accession #1439** | **Benign**  **(n=6)** | **Primary Prostate Cancer**  **(n=7)** | **Metastatic Prostate Cancer**  **(n=6)** |
| --- | --- | --- | --- |
| SSX2 | 0.0 | 0.0 | 436.9 |
| CSAG2 | 0.0 | 0.0 | 1018.2 |
| MAGEA2 | 0.0 | 0.0 | 229.0 |
| MAGEA12 | 0.0 | 0.0 | 1139.7 |
| CEP55 | 0.0 | 56.0 | 640.2 |
| NUF2 | 14.1 | 32.3 | 1788.6 |
| PBK | 30.1 | 120.3 | 1411.5 |
| TTK | 83.8 | 216.0 | 1187.1 |
| PAGE4 | 6732.5 | 5702.8 | 0.0 |
| TBP | 608.7 | 577.6 | 882.2 |
| ACTB | 42572.0 | 41320.7 | 38735.7 |

GEO; The Gene Expression Omnibus (<http://www.ncbi.nlm.nih.gov/geo>)
