# Supplementary material for: Cancer/Testis antigens as potential predictors of biochemical recurrence of prostate cancer following radical prostatectomy
Source: J Transl Med. 2011 Sep 14;9:153. doi: 10.1186/1479-5876-9-153 (PMC3184272; doi:10.1186/1479-5876-9-153)
Supplement: Additional file 2 — Supplemental Table 2. PCR primer and probe sequences for quantitative singleplex and multiplex real-time PCR. This file contains PCR primer and probe sequences used in this study. [file 1479-5876-9-153-S2.DOC]

Supplemental Table 2. PCR primer and probe sequences for quantitative singleplex and multiplex real-time PCR

| **Gene** | **Forward primer sequence** | **Reverse primer sequence** | **Probe sequence** | **Dye** |  |
| --- | --- | --- | --- | --- | --- |
| **TBP** | GAATATAATCCCAAGCGGTTTG | ACTTCACATCACAGCTCCCC | CACAGGAGCCAAGAGTGAAGAACA | FAM | Set1 |
| **GSTP1** | AGGGAGGCAAGACCTTCATT | GGCTAGGACCTCATGGATCA | CTCCTTCGCTGACTACAACCTGCT | Cy5 | Set1 |
| **NUF2** | TGCCGTGAAACGTATATGGA | ATTAATGCCTCCTGGTGTGC | TTTGGCAATATAAATCCTCTGCGG | TEX-RED | Set1 |
| **PBK** | TCTCATTCTCCTTGGGCTGT | TGCCATCATTGGCTTCAGTA | TCCAAACATTGTTGGTTATCGTGC | HEX | Set1 |
| **ACTB** | CCTGGCACCCAGCACAAT | GCCGATCCACACGGAGTACT | AGATCAAGATCATTGCTCCTCCTGA | FAM | Set2 |
| **PAGE4** | CGTAAAGTAGAAGGTGATTG | ATGCTTAGGATTAGGTGGAG | GGATCTGGAAAAGACTCGGAGTGA | Cy5 | Set2 |
| **TTK** | CAGCAGCAACAGCATCAAAT | TGCTTGAACCTCCACTTCCT | CAGCAAATGAATGCATTTCGGTTA | TEX-RED | Set2 |
| **CEP55** | ACTGTGGCTCCAAACTGCTT | GAGCAGCTGTTTCCGTTTTC | TGCTCTGGAGAAAAATCAGCAGTG | HEX | Set2 |
| **SSX2/3** | GGTGGAGCAGTCAGAACACA | TGGGTCCCTGTTGTGTGTAA |  |  |  |
| **CSAG2** | AGTGGGCCAACACTATCCAG | CTGGCTGTCCGAAGAGAGAC |  |  |  |
| **MAGEA2** | CAGCAACCAAGAAGAGGAGG | TGCAAGTACTCGGAGGCTTT |  |  |  |
| **MAGEA12** | CTGGAGTCAATCCGATGAGG | TCTCCAGGTCAGGAAAGGTG |  |  |  |
